# MoMuLV-ts-1: A Unique Mouse Model of Retrovirus-Induced Lymphoma Transmitted by Breast Milk

**DOI:** 10.1155/2011/813651

**Published:** 2011-08-16

**Authors:** J. Chakraborty, H. Okonta, H. Bagalb, J. Duggan

**Affiliations:** ^1^Department of Physiology and Pharmacology, Block Health Science Building, College of Medicine, University of Toledo, Health Science Campus, Toledo, OH 43614, USA; ^2^Department of Medicine, Ruppert Building, College of Medicine, University of Toledo, Health Science Campus, Toledo, OH 43614, USA

## Abstract

Our laboratory has developed a murine model of lymphoma via breast milk transmission of MoMuLV-ts-1 (Moloney murine leukemia virus-temperature sensitive mutant-1). Uninfected offspring suckled from infected surrogate mothers become infected and develop lymphoma. Multiple gene integration sites of ts-1 into the infected mouse genome including *tacc3, aurka, ndel1, tpx2, p53, and rhamm* were identified, and mRNA expressions were quantitated. These genes produce centrosomal proteins, which may be involved in abnormal chromosomal segregation leading to aneuploidy or multiploidy, thus causing lymphoma. Since there is no report to date on this retroviral model leading to centrosomal abnormality, and causing lymphoma development, this is a valuable and unique model to study the centrosomal involvement in lymphomagenesis.

## 1. Introduction

Retroviral infections in humans range from asymptomatic murine leukemia virus infections to deadly infections such as HIV. The HIV epidemic has spurred unprecedented levels of retroviral research in the last three decades due to the ever-increasing human toll. According to the WHO and UNAIDS report in 2008 [1], globally 33.4 million people are now living with HIV. Of these, 2.1 million children under the age of 15 years are suffering from HIV infection and 280,000 children died of AIDS and AIDS-related conditions such as lymphoma in 2008. In 2008, an additional 430,000 children became infected, with the vast majority living in developing countries with little access to antiretroviral therapy (ART). The main route of infection in these children is mother to child transmission (MTCT). Although treatment of infected mothers during pregnancy with ART and use of infant formula after delivery has limited MTCT of HIV-1 in developed countries [1], these options are often unavailable in Africa and other developing countries where MTCT still remains a significant source of HIV infection.

Due to practical and ethical constraints involving human subjects, the mechanism of perinatal transmission of HIV-1 is not yet fully understood. HIV can be transmitted to the fetus/infant during prenatal and postnatal periods. A suitable animal model may allow in depth study of the pathophysiologic mechanisms of MTCT of HIV. We have successfully developed a murine model for mother-to-offspring transmission of Moloney Murine Leukemia virus (MoMuLV) ts-1, a retrovirus which mimics HIV. We have clearly demonstrated that the transmission of ts-1 can occur in utero, intrapartum, and/or postpartum. Transmission of ts-1 produces an immunodeficiency state with wasting, increased infection, and neurologic deterioration. In addition, postpartum transmission of ts-1 is associated with an increased development of lymphoma in pups [2, 3]. 

Chakraborty et al. [4] have found that ts-1 integration leads to an overexpression of four genes associated with lymphoma in BALB/c mice infected by breast milk. This unique model has allowed us to demonstrate ts-1 retrovirus transmission via breast milk and study the molecular mechanism of the lymphoma development through natural transmission of a retrovirus to the offspring. Our hypothesis is that viral integration into the host genome alters host gene expression leading to an abnormal mRNA expression and abnormal protein production. These abnormal proteins alter the centrosome function during the cell cycle resulting in lymphomagenesis. This paper will explore two related areas of ts-1 research: ts-1 as a small animal model of perinatal retroviral transmission and ts-1 in lymphomagenesis.

## 2. Part I. MoMuLV-ts-1 as a Small Animal Model

### 2.1. The Similarities and Differences between HIV and MoMuLV-ts-1

HIV shares many characteristics with ts-1. These include CD4 cell targeting, secondary infections, neurodegenerative diseases, macrophage and CD4 cell infection, immunodeficiency, neurotropism, CD4 cell depletion, wasting, lymphomas, and perinatal transmission. However, the mechanism of entry of these two viruses are different. The Moloney murine leukemia virus (MoMuLV) ts-1 is a temperature sensitive mutant virus [5–7] first isolated by propagating MoMuLV in a thymus bone marrow cell line (TB) taken from CFW/D mice. This virus has a defect in the intracellular processing of the envelope precursor polyprotein (Pr80env) at the restrictive temperature [6–9]. Like HIV, MoMuLV ts-1 infects CD4 cells, with subsequent CD4 depletion and a resulting immunodeficiency (see Table 1) [10]. ts-1 is a murine gamma retrovirus that can induce T-cell lymphomas in susceptible strains of mice after a long incubation period [11].

As a simple retrovirus, ts-1 has only three genes (gag, env, and pol). Infection with ts-1 results in an AIDS-like syndrome in mice similar to HIV infection in humans [10, 12–14]. The most important characteristic of ts1 to our study is its effects on MTCT by breastfeeding in mice [2–4]. About 97% of uninfected neonatal mice that suckle from an infected mother develop clinically symptomatic ts-1 infection [3, 4]. Newborn BALB/c mice infected with ts1 virus suffer from neurodegenerative disease resulting in hind limb paralysis and immunologic disease characterized by severe thymic atrophy associated with immunodeficiency due to destruction of T-lymphocytes, and generalized body wasting [7, 15]. Infectivity of ts-1 is significantly related to its temperature sensitivity [7], and can replicate optimally at a permissive temperature of 34°C [16]. This may explain why the ts-1 virus can produce hind limb paralysis in newborn mice and not in the adult, because the body temperature of the newborn mice is lower (~34°C) than that of adult (38.4°C) [17]. The uniqueness of the ts-1 among other murine retroviruses is that it can cause degenerative diseases in mice similar to HIV in humans, by affecting both the central nervous system (CNS) and the immune system. Infected T-lymphocytes have impaired function [14, 18]. 

The murine ts-1 model has been extensively used as a small-animal model for retrovirus-induced neurodegenerative disease [19]. Oxidative stress has been suggested as a major mechanism for ts1-induced neurodegeneration and T-cell loss in infected newborn mice [20]. The U3 region of ts-1 controls the pathogenicity and targets cell type [21]. 

Transfer of humoral immunity to ts-1 can be passed from mother to baby via breast milk and can provide protection from neurodegenerative and immunologic disease in neonatal mice [22]. Chakraborty et al. [23], have further developed this murine model to study the perinatal transmission of ts-1. Infected mothers can transmit the ts-1 virus vertically to offspring. They further demonstrated that mother-to-offspring transmission via breast milk can occur at nearly a 100% incidence [4], and can cause lymphoma when control pups suckle from ts-1 infected mothers. The pattern of proviral ts-1 integration sites observed in these lymphoma tissues correlates with the upregulation of mRNA expression of candidate genes that may contribute to lymphomagenesis [4].

### 2.2. Mother-to-Child Transmission of HIV by Breastfeeding

MTCT of HIV can occur *in utero, intrapartum,* or through breastfeeding. Current strategies aimed at decreasing MTCT of HIV have focused mainly on *in utero* and *intrapartum *transmission. Prophylaxis with highly active antiretroviral therapy (HAART) during pregnancy and through delivery has substantially reduced the rate of MTCT of HIV at birth [24]. Avoidance of breastfeeding by HIV-infected mothers is the norm in developed countries throughout the world [25]. However, for socioeconomic and cultural reasons, such avoidance is not an acceptable or viable alternative for many HIV-infected women in Africa and other resource poor areas worldwide [26]. In these areas, breastfeeding is the best and only source of infant nutrition, and safe alternatives do not exist because of inadequate supplies of formula and the lack of clean water. Breastfeeding, therefore, accounts for a substantial proportion of the overall MTCT rate in developing countries. Breastfeeding for 15 months accounts for 38% of the overall MTCT rate [27], and if continued for 24 months, it accounts for 44% [28]. Richardson et al. [29] reported that if an infant ingests one liter of breast milk then the probability of having the HIV-1 infection is similar to that of heterosexual transmission. Breast milk transmission depends on high maternal viral load and status of immunodeficiency with mastitis and duration of breastfeeding increasing the rate of HIV infection [29]. Breastfeeding by HIV positive women can reverse the benefits made in reducing perinatal HIV transmission through the use of HAART during pregnancy and/or labor. For example, in the Petra study of MTCT of HIV [30], the benefits of perinatal HAART prophylaxis were lost in the breastfeeding group. In order to prevent milk-borne HIV, HAART must be given throughout the breastfeeding period, which is rarely a feasible option in developing countries.

Studies in primates with HIV-2 [31], simian immunodeficiency virus (SIV) and SHIV (SIV expressing HIV envelope) [32, 33] have shown that primate lentiviruses can be transmitted orally. After milk is swallowed, gastric acid may inactivate some virus, but the buffering capacity of milk may allow the remaining virus to enter into the lumen of the gut, where there is extensive lymphoid tissue as a target for HIV. Mixed feeding (breastmilk and formula) carries a significantly increased risk of HIV transmission, because formula foods increase gut permeability [34]. These data suggest that the intestine could be a site of HIV entry in breast milk transmission, in addition to the oropharyngeal cavity. In areas without clean water, WHO recommends exclusively breastfeeding to avoid fatal diarrhea and the increased risk of HIV infection related to mixed feeding of formula and breast milk [35].

Several methods have been studied to decrease MTCT of HIV in areas where breastfeeding is a necessity. These include wet nursing by an HIV-negative woman, heat-treating the mother's milk with Holder pasteurization (62.5° for 30 min), leaving milk at room temperature for 30 minutes to allow milk lipase to inactivate HIV, adding microbicides to milk and standing for 5–10 minutes, or getting milk from a human milk bank [35, 36]. Due to high cost, obtrusiveness, inaccessibility, and/or inconvenience, none of these options are practical in developing countries where breastfeeding remains the only viable option for infant feeding and thus a major route of vertical HIV transmission. Therefore, a prophylaxis is urgently needed. However, in order to develop an effective prophylaxis, the exact mechanism of breast milk transmission of retrovirus must be clearly determined. In-depth study of the pathophysiology of retroviral transmission via breast milk using an animal model such as our ts-1 model will provide mechanistic insights that can translate into effective human therapies.

### 2.3. Breast Milk Transmission of MoMuLVts-1 in BALB/c Mice

In the study of viral transmission by breast milk, use of a small animal model with a well-characterized immune system, such as the mouse, is ideal. As mentioned above, infection with ts-1 results in an AIDS-like syndrome. When neonatal BALB/c mice less than 10 days old are injected intraperitoneally with ts-1, clinically apparent infection develops within 10 weeks. This clinical syndrome in mice is characterized by progressive bilateral hind limb paralysis, severe body wasting, and immunodeficiency [15]. Milder clinical illness can be induced by varying the dose of virus injected and/or the time of viral inoculation up to 10 days of age. CD4 cells are depleted by apoptosis, yielding AIDS-like clinical manifestations [12–14].

Several unique characteristics of this virus determine the experimental manner in which it is evaluated. The infectivity of this virus is strain specific and dependent upon the age of the mouse at the time of exposure, but the temperature sensitive nature of the virus does not affect its *in vivo *infectivity [15, 37]. BALB/c mice are a susceptible strain, with clinical diseases developing in 98% of mice by week 10 after intraperitoneal injection. Viral injection of mice after ten days of age does not result in clinically detectable disease but does result in antibody production [19, 38]. Perinatal transmission of ts-1 occurs *in utero, intrapartum*, and via breastfeeding [2, 3]. Breastfeeding is a highly effective route of MTCT in this model: 95% to 99% of uninfected neonatal mice, if suckled from an infected mother, develop clinically symptomatic ts-1 infection [3, 4].

**Figure 1:**
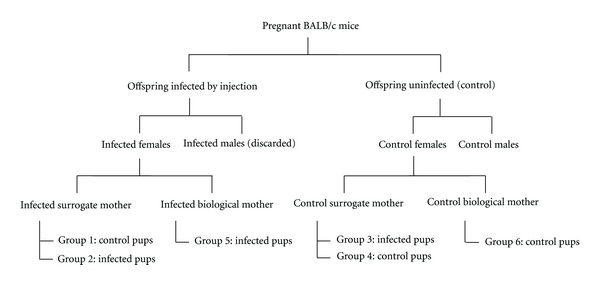
Experimental plan for ts-1 breast milk transmission studies.

Our laboratory was the first to clearly demonstrate that breast milk transmission in offspring of ts-1 infected BALB/c mice causing AIDS like condition. We have established a mouse model for MTCT for MoMuLV ts-1 for in utero, intrapartum, and postpartum (breastfeeding) routes of transmission [2–4, 23]. The following information provide some murine model data for retroviral transmission via breastfeeding. Pregnant BALB/c female mice delivered pups which were divided into experimental and control groups. Seventy two hours after birth, the pups were injected intraperitoneally (ip) with 0.1 mL of 4.0  ×  10^6^ ffu/mL ts-1 virus and control pups were injected with 0.1 mL DMEM medium [2–4]. These mice were allowed to mate with uninfected males and produce offspring. Within 10–12 h after birth, offspring suckled either from control or ts1-infected surrogate or biological mothers (Figure 1). Tissue collection, histology, electron microscopy, flow cytometry, DNA extraction, PCR, and sequencing of viral DNA were performed. Four hundred twenty one mice were divided into 6 groups as described in our previous paper [3]. Pups from the ts-1 control mothers suckled from the infected surrogate mothers (Group 1), pups from the infected mothers suckled from the infected surrogate mothers (Group 2), or from infected biological mothers (Group 5). In Group 3, infected pups suckled from the control mothers. Groups 4 and 6 were controls. Group 4 control pups suckled from the control surrogate mothers, and Group 6, control pups suckled from their control biological mothers.

**Figure 2:**
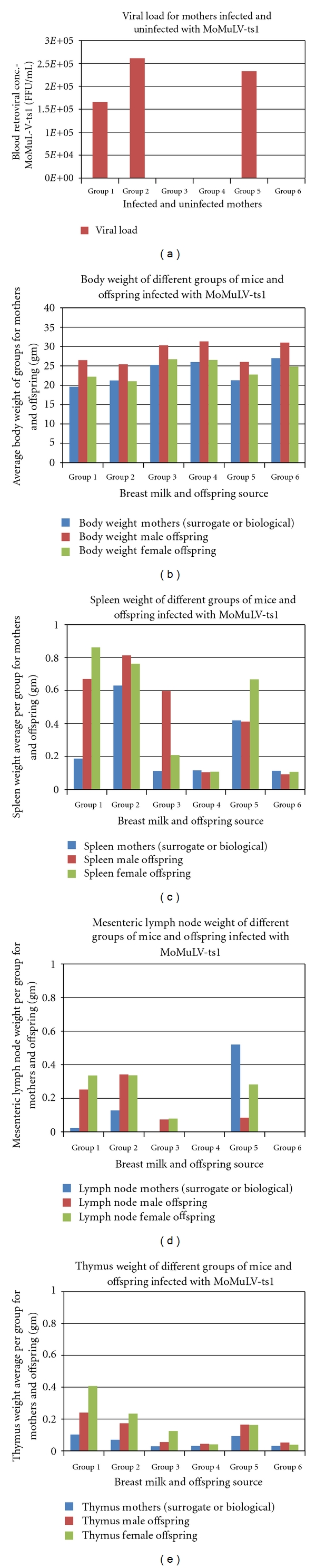
Viral load comparisons of infected and control mothers. Body, spleen, lymph node, and thymus weight comparisons for mothers and offspring of different groups.

**Figure 3:**
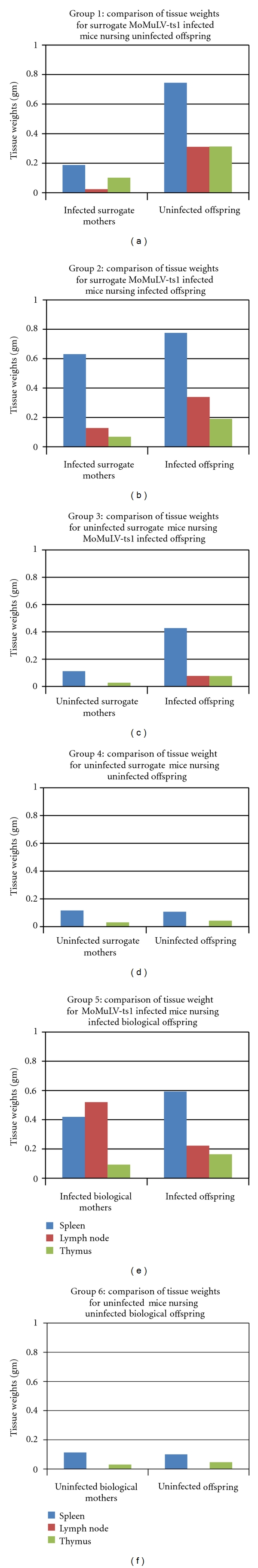
Tissue weight of mothers and offspring of each of 6 groups.

**Figure 4:**
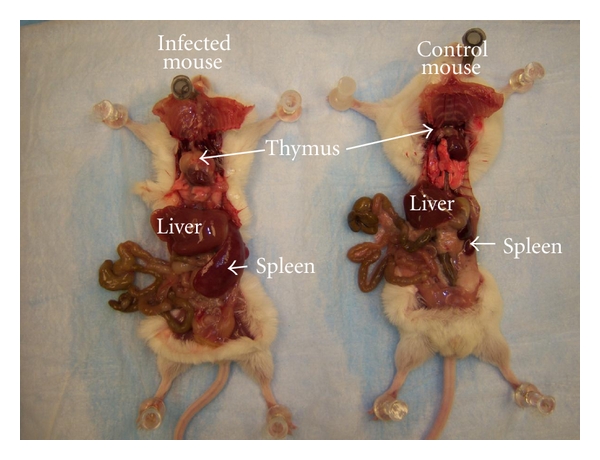
Organomegaly in infected versus control mice.

**Figure 5:**
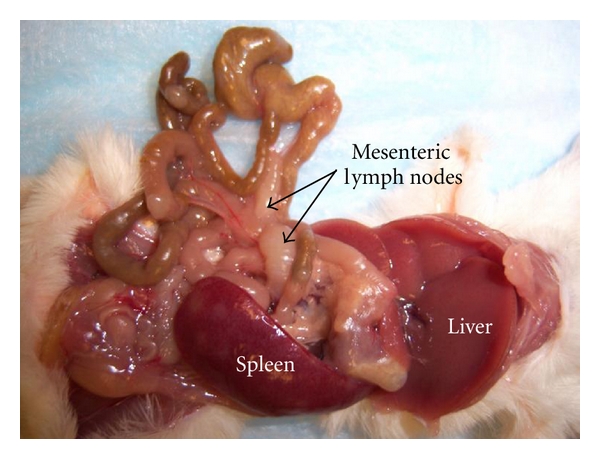
Manifestations of MoMuLV-ts-1 retroviral infection on different organ system.

**Figure 6:**
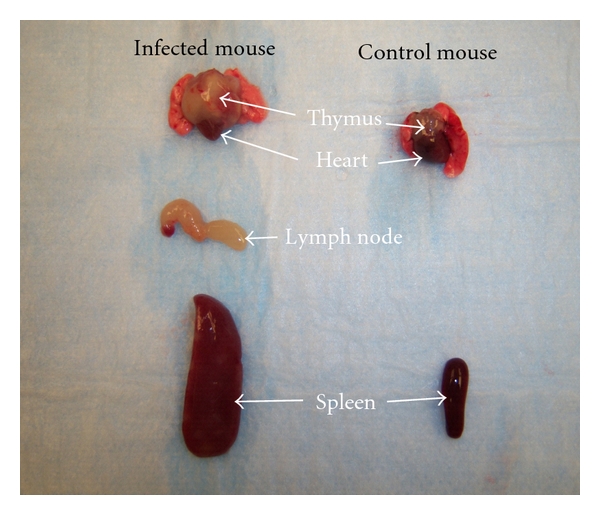
Excised organs from infected versus control mouse.

**Figure 7:**
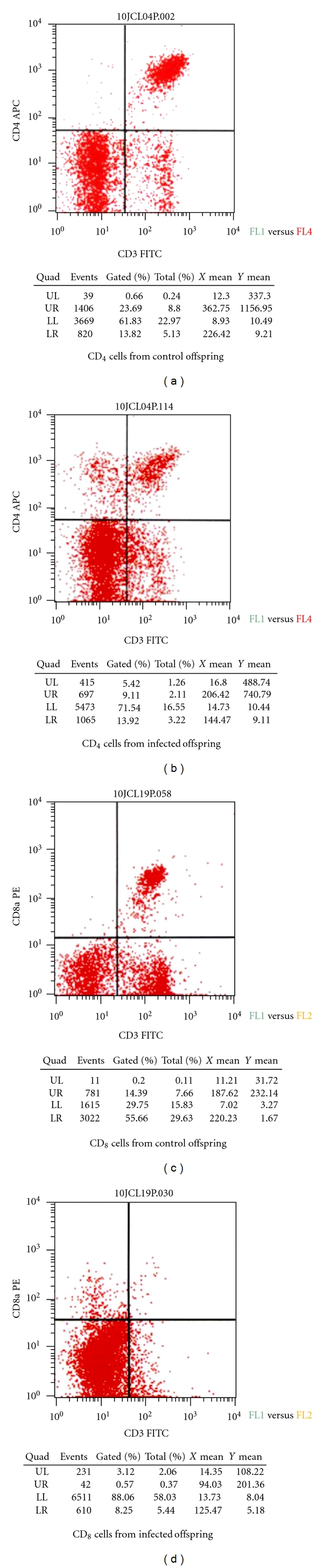
Examples of the flow cytometric analysis showing frequency of CD4+ and CD8+ cells from control and infected offspring. Samples were tagged with CD3 FITC, CD4 APC and CD3 FITC, CD8 PE. Upper left quadrant (UL) shows naïve lymphocytes. Upper right quadrant (UR) shows CD3+/CD4+ or CD3+/CD8+ frequency for cells positive for both CD3 and CD4 or CD3 and CD8 markers. Lower left quadrant (LL) shows CD3−/CD4− or CD3−/CD8− frequency for cells negative for both CD3/CD4 or CD3/CD8 markers. Lower right quadrant (LR) shows cells positive for CD3 markers only.

The rate of postpartum ts-1 transmission in this study was almost 100%. This rate is much higher than the MTCT of HIV in infants via breastfeeding (about 20%–30%), which is advantageous in an experimental animal model. Maternal viral load appear to correlate with development of lymphoma (Table 3; Figures 2 and 3), but further studies are needed to verify this. The figures and tables listed below show some of the gross morphology and flow cytometric data. Significant and consistent increases in weights and change in gross morphology of spleen, lymph node, and thymus (Tables 2 and 3; Figures 4, 5, and 6) are readily apparent. Our flow cytometric data shows decreases in CD4 and CD8 (Figure 7) cell population in the advanced stages of lymphoma development. In addition, decreasing numbers of CD8 and B-cells have been shown (Table 4) in the presence of clinically apparent immunodeficiency and wasting.

## 3. Part II. ts-1 and Lymphomagenesis

### 3.1. Retrovirus Induced Cancer

The first reports of retroviruses associated with cancer occurred in the early 20th century when an avian erythroblastosis virus (AEV) was isolated from spontaneous erythroleukemia in a chicken [39]. Shortly after that, Peyton Rous demonstrated that chicken sarcomas were infectious and could induce tumors when transmitted into healthy birds [40]. These novel observations were followed by multiple reports of retroviruses isolated from a broad range of mammals such as rodents, cats, sheep, and cows in association with both malignancies and immunodeficiencies. The first human retrovirus, human T-lymphotropic virus type 1 (HTLV-1), was isolated in 1980 [41] and has been shown to induce adult T-cell leukemia (ATL) [42].

Retroviruses are classified into seven genera. Oncogenic retroviruses (retroviruses that induce tumorigenesis) belong to the following genera (Table 5) [43, 44].

**Figure 8:**
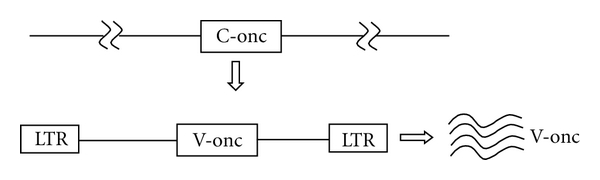
Acute transforming virus, capture of a c-onc and over-expression of v-onc by provirus.

Oncogenic retroviruses which induce tumors can be divided into two classes: acute and slow transforming viruses. Acute transforming retroviruses induce polyclonal tumors within 2 to 3 weeks after infection of the host. These retroviruses induce tumors through acquisition and overexpression of cellular proto-oncogenes that have captured virus (Figure 8) [45]. An example is v-Abl in the Abelson Murine Leukemia Virus [46].

**Figure 9:**
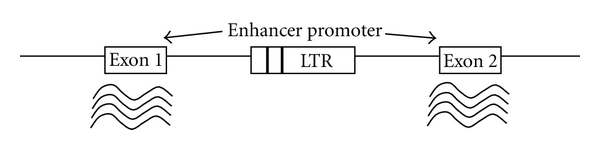
Enhancer of promoter insertion either upstream or downstream of growth controlling cellular genes.

In contrast, slow transforming retroviruses induce mono- or oligoclonal tumors with a longer latency of several months. These types of retroviruses do not carry viral oncogenes and can cause tumors by activating cellular proto-oncogenes close to the proviral DNA integration site on the host genome (Figure 9).

Elements in the proviral genome that regulate the viral transcript also act at common integration sites (*cis)* on cellular gene transcripts. Depending on whether the provirus integrates into the genes or in the vicinity of the genes, these elements can enhance or disrupt normal transcription, and thus induce oncogenic mutations. These classes of retroviruses have been found to induce tumors in many animals, including birds (ALV and REV), and mice (MLV); [45]. In addition to these two general transformation groups, a small number of retroviruses induce tumors by expression of their own oncogenic proteins. For example, human T-cell leukemia virus types 1 and 2 (HTLV-1 and HTLV-2, resp.) induce adult T-cell immortalization and leukemia in human by expression of viral Tax protein. Tax has no cellular homologue, and it works in trans to disrupt cellular checkpoints and destabilize genome integrity [47] leading to transformations that directly cause human cancer [48]. In AIDS-related lymphoma, patient studies indicate that oncogene activation by insertional mutagenesis of the HIV might be another mechanism by which HIV-1 can induce cancer [49, 50]. 

The release of the complete mouse genome sequence and the availability of reliable methods for isolation of proviral flanks have introduced the retroviral insertion mutagenesis screen in mice as a powerful procedure to identify genes contributing to tumorigenesis. Many oncogenes identified in these screens have given a valuable basis for better understanding the development of human cancer [45]. The MLV utilizes a slow transformation mechanism to induce leukemia or lymphoma in mice and is one of the retroviruses that provides an excellent model to identify and study the oncogenes involved in retrovirus-induced tumorigenesis.

### 3.2. Centrosomal Involvement in Cell Cycle and Cancer

**Figure 10:**
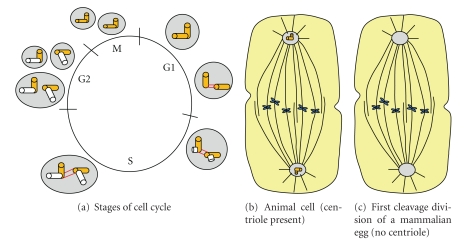
This figure shows the centriole-centrosome division during cell cycle (a), presence of both centriole and centrosome in the animal cell (b), and absence of centriole in the first cleavage division of a mammalian egg (c).

Centriole-centrosome is the microtubular organization center (MTOC) and plays a major role during mitosis for chromosomal arrangement at the equatorial plane during metaphase and pulling the chromatids in two opposite spindle poles during anaphase. Spindles start to organize as soon as centrioles divide and two centrosomes are formed. The spindle microtubules act like railroad tracks on which the chromatids travel towards the poles with the help of several motor proteins including dynein and kinesin. However, the centriole is not an essential structure for many cells including first cleavage division of mammalian eggs [51].  Figure 10 describes the centrosome-centriole cycle in a typical animal cell at mitosis and the first cleavage division of a mammalian egg without the centriole. The centrosome—which is an accumulation of amorphous materials around the centriole—is present in most mammalian cells. In these cells, the centrosome is an essential component in the development of spindle poles and progression to mitosis and cytokinesis. There are three types of spindles: kinetochore, interpolar, and astral. The kinetochore spindle extends from poles to chromosomes. The interpolar spindles run from pole to pole with no connection to the chromosomes, while the astral spindles run between centrosomes to the cell cortex. Thus, three types of spindles play different roles. 

**Figure 11:**
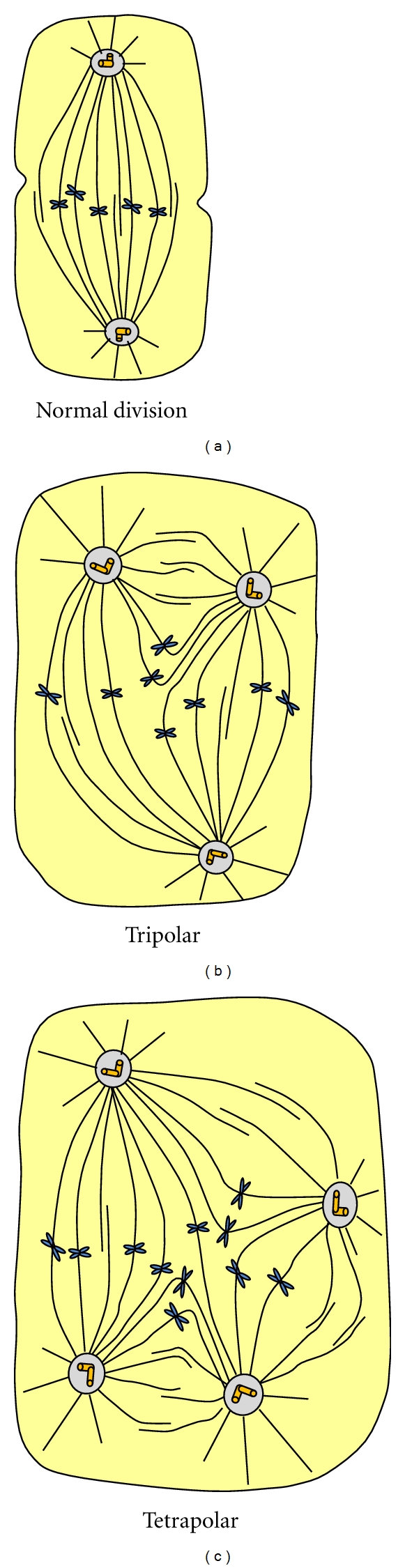
Normal and abnormal cell division due to abnormal centriole-centrosome division. As a result, giant cells with multiple nuclei may be present in a malignant cell.

There are numerous centrosomal proteins and new proteins are being discovered every year. Although the centriole-centrosome system is essential for cell cycle and normal chromosomal segregation, little is known about the intricate details of this structure, which is extremely important in the understanding of cancer development and progression. For example, many cancers show abnormal mitosis leading to tetraploidy and aneuploidy. According to Wang et al. [52], many carcinomas may have either or both structural and numerical abnormalities; this occurs during early stage of tumorigenesis. This is also correlated with chromosomal instability and tumor progression. Numerical chromosomal abnormalities can also occur due to bipolar and tripolar or tetrapolar spindle formation (Figure 11).

Unequal segregation of chromosomes leads to multiploidy and/or aneuploidy. Viral gene insertion into the normal genome close to the centrosomal genes may lead to abnormal mRNA expression resulting in abnormal protein production and activity. For example, increases in *Tacc3* and *Aurora A kinase* (Aurka) can produce tumor like cells [53, 54].

### 3.3. Insertion of Viral Genome into the Mouse Genome Causing Abnormal Centrosomal Protein Production Leading to Lymphomagenesis

**Figure 12:**
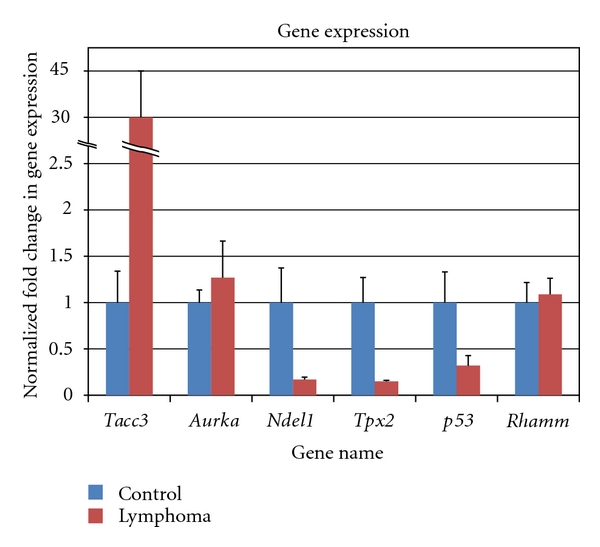
Graph of fold change in gene expression versus normalized control.

A large number of investigations have been carried out to study the development of lymphoma by MoMuLV, but so far, little data are available on the natural transmission of this retrovirus by breast milk causing lymphoma. Extensive studies are needed on breast milk transmitted retroviruses, since evidence exists that viruses such as HTLV-1 or Epstein-Barr virus (EBV) may be oncogenic and also have epidemiologic patterns indicating perinatal or early childhood transmission. The best studied of these viruses is EBV. Studies have shown that EBV and MLV gene insertion into the host genome alters gene expression leading to carcinogenesis [55–59]. We have used inverse PCR (I-PCR), DNA cloning, sequencing, and quantitative reverse transcriptase-PCR (qRT-PCR) to study the viral integration into mouse genome. Tissue samples from spleen and lymph nodes were used for the genomic studies [4]. Using the primary PCR product as template, secondary PCR was performed. After the PCR bands were obtained, cloning was done and colonies with viral inserts were located and analyzed. The differential expression of candidate genes was obtained by using quantitative real-time PCR. Gene specific primers were designed using Primer Express software. GAPDH was used as “housekeeping” gene to confirm amplification factor for each PCR product melt-curve analysis process. RNA expression levels were calculated by using ddCt method. Gene expression were recorded by mRNA expression and reported as “fold change”. Statistical analyses were performed by using statistical package of the social sciences (SPSS). 35 genes were selected for mRNA expression based on preselected criteria.  Table 6 and Figure 12 show the change in mRNA expression of six genes *Tacc3* shows the highest upregulation with 30.13-fold increase, much more than our previous report [4].

**Figure 13:**
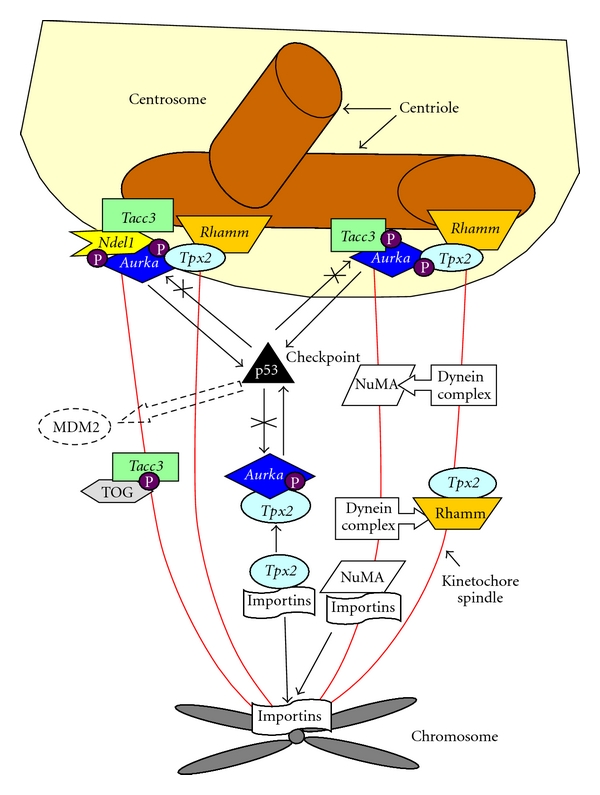
Pathway of Genes associated with spindle formation.

How are changes in gene expression associated with lymphoma in this murine model? In our mouse model, about 50% of pups develop lymphoma. This high incidence of lymphoma may be due to the genomic integration of viral DNA into the mouse genome causing changes in nearby genes. While viral gene insertion appears to be random, in pups that develop lymphoma, there is a predilection for viral insertion near genes involved in spindle formation. One of the mechanisms of lymphoma development, therefore, may be the alteration in spindle assembly-disassembly pathway. Up or down regulation of these genes may occur, breaking the balance of protein production, and causing abnormality in spindle formation, chromosomal segregation, and subsequent lymphoma production. Although there are many genes involved in the spindle assembly pathway, we have studied 6 genes predominately associated with viral genomic integration in mice with lymphoma including *tacc3, aurka, tpx2, rhamm, ndel1 *and* p53*. Of these 6 genes, we have observed the highest fold increase of *tacc3* followed by *aurka*.  Figure 13 shows the proteins (gene product) associated with spindle formation.

All tumor samples showed increases in mRNA expression of *tacc3 * gene compared to the control group. Out of the 6 genes reported in this study, two were upregulated, including* tacc3 *and* aurka. *As previously reported, *tacc3* showed the highest mRNA expression levels with an average of 9.16-fold increase [4]. *tacc3* is associated with centrosome and microtubule-associated proteins that are essential for mitotic spindle formation [60] and have been associated with dysfunction in a variety of tumors. *Tacc3* was also identified as a novel prognostic marker in nonsmall cell lung cancer [61]. Dysregulation of T*acc3* proteins have also been related to ovarian cancer [62]. Schnieder et al. [63] demonstrated the important role of T*acc3* in spindle assembly and cellular survival, thus introducing it as a potential therapeutic target in cancer cells. Deficiency of *Tacc3* leads to *p53*-mediated apoptosis [64]. Therefore, overexpression of *tacc3*, as observed in our mouse model may cause the downregulation of *p53*, causing inhibition of apoptosis, thus leading to lymphoma.

*Aurka* connects microtubules to kinetochore and phosphorylates T*a*c*c3,* leading to its localization to spindle microtubules during metaphase and promoting its growth from centrosomes. *Aurka* is essential for accurate chromosome segregation. Overexpression of *Aurka* leads to spindle defects, aneuploidy, and tumor formation. *Tpx2* and *Rhamm* helps with stable bipolar spindle formation through its association with *Aurka* [65–67]. Decrease in expression of these proteins may cause spindle fragmentation, while increased expression may cause disorganized, multipolar spindles coupled with inability to appropriately align and segregate chromatids. *Ndel1* has a high affinity for T*acc3,* and its disruption decreases centrosome targeting of T*acc3 *[68].* p53* is a cell-cycle checkpoint protein. DNA damage generally results in phosphorylation of *p53* leading to its dissociation from *MDM2* and acts as a transcription factor, arresting the cells in the G1 phase of the cell cycle. Mutated or downregulated *p53* results in continued proliferation in damaged cells and subsequent development of cancer. Upregulation of *aurka* and *tacc3* and downregulation of *p53* are consistent with abnormal cell division, aneuploidy, and tumorigenesis. Downregulation of *tpx2* is associated with abnormal spindle formation with the presence of giant cells and the absence of mitotic figures in histological specimens of lymphoma tissue. However, spindle pathway disruption is not the only mechanism by which malignancies develop or progress, but it may be an important factor for breast milk-transmitted retrovirus-induced cancer in the ts-1 mouse model.

## 4. Conclusion

A well-developed animal model can be extremely important in the study of viral-induced cancer and immunodeficiency. Although there are obvious differences between murine and human cells, an appropriate mouse model can provide important clues to the possible pathways of molecular mechanisms of retroviral transmission and cancer development and progression. While this paper reviews ts-1 as a small animal model of perinatal HIV transmission and ts-1 in lymphomagenesis, it also raises a number of questions that need further study. We believe that this is a unique murine model for the study of retrovirus-induced lymphomagenesis for multiple reasons. Mice have been used extensively for genetic, physiological, biochemical, immunological, endocrinological, and reproductive research and an enormous body of background data—including the entire murine genome—is already available. In our unique murine model, breast milk transmission is near 100%, but lymphoma development occurs in only 50% of infected pups across all litters; therefore, an ideal internal control exists in which to study causative genetic factors. Study of this model may contribute to the understanding of molecular mechanisms of spindle formation, chromosomal segregation, and interactions between many centrosomal proteins and may help clarify some of the cellular abnormalities leading to cancer. Some of these proteins and their inhibitors can be used for diagnostic and/or therapeutic development.

## Figures and Tables

**Table 1:** Comparison between HIV and MoMuLV-ts-1 showing the similarities and differences between these two types of viruses.

Characteristics	HIV	ts-1
Retrovirus	+	+
Lentivirus	+	−*
Initial target for infection: CD4 cells	+	+
Depletion of T-lymphocytes	+	+
T-cell death via apoptosis	+	+
Inefficient transport of env precursor	+	+
Viral induced cytokine production	+	+
Polyclonal activation of B lymphocytes	+	+
Infection of astrocytes and microglia	+	+
Demyelination	+	+
Neuronal death	+	+
Wasting	+	+
Can cause lymphoma in some cases***	+	+
Horizontal transmission	+	−**
Mother-to-offspring transmission	+	+
Can cause disease	New born and adult	New born only

*MoMuLV-ts-1: mammalian gamma oncoretrovirus. **ts-1: No adult mouse can be infected; therefore, no sexual transmission occurs. ***: Both HIV and ts-1 may indirectly cause lymphoma.

**Table 2:** Percentages of changes in spleen, lymph node, and thymus due to MTCT of ts-1 virus by breastfeeding in control and infected pups.

Group	Description	Sample size (*n*)	Splenomegaly	Lymph node hypertrophy	Thymic hypertrophy
1	Control pup nursed by infected surrogate mother	93	95.7%	79.1%	95.7%
2	Infected pup nursed by infected surrogate mother	94	92.6%	56.5%	92.6%
3	Infected pup nursed by control surrogate mother	108	46.3%	25.9%	46.3%
5	Infected pup nursed by biological mother	88	89.8%	61.4%	89.8%
4 & 6	Control—no infection	154	1.9%	0.0%	5.8%

**Table 3:** Correlation between mothers viral load and selected pup body and organ weights.

Group	Mothers					Pups			
	Treatment	Sample size (*n*)	Viral load	Body weight	Sample size (*n*)	Body weight	Spleen weight	Lymph node weight	Thymus weight
1	Infected surrogate mother/control pup	6	1.657*E*+05	19.58	32	24.14	0.74	0.31	0.31
2	Infected surrogate mother/infected pup	11	2.612*E*+05	21.24	57	23.44	0.78	0.34	0.19
3	Uninfected surrogate mother/infected pup	6	0.000*E*+00	25.24	38	28.40	0.43	0.08	0.08
4	Uninfected surrogate mother/infected pup	15	0.000*E*+00	25.99	80	28.31	0.11	0.00	0.04
5	Infected biological mother	17	2.332*E*+05	21.25	66	24.18	0.59	0.22	0.16
6	Uninfected biological mother	15	0.000*E*+00	27.00	81	28.44	0.10	0.00	0.05

Total number of mothers: 67; total number of offspring males: 158; total number of offspring females: 196; total number of animals 421.

**Table 4:** Flow cytometric Analysis of CD4+, CD8+, and CD19 Cells.

Flow Cytometric analysis				
		CD4 (T-cell)	CD8 (T-cell)	CD19 (B-cell)
Blood	Control	15.4 ± 10.6	4.4 ± 2.9	2.6 ± 1.2
	Infected (no lymphoma)	5.1 ± 4.2	1.0 ± 0.7	0.5 ± 0.6
	Infected (with lymphoma)	3.2 ± 2.4	1.5 ± 1.2	1.7 ± 1.3
Spleen	Control	13.1 ± 4.7	4.9 ± 2.0	22.6 ± 7.1
	Infected (no lymphoma)	12.5 ± 0.8	4.6 ± 0.3	10.1 ± 2.4
	Infected (with lymphoma)	2.8 ± 0.6	1.8 ± 1.2	2.2 ± 1.2
Thymus	Control	15.6 ± 5.6	16.6 ± 8.5	0.9 ± 0.2
	Infected (no lymphoma)	25.3 ± 33.3	1.2 ± 0.1	2.0 ± 1.4
	Infected (with lymphoma)	8.6 ± 9.2	8.9 ± 13.5	0.4 ± 0.5
Lymph Node	Control	(NA)	(NA)	(NA)
	Infected (no lymphoma)	(NA)	(NA)	(NA)
	Infected (with lymphoma)	11.3 ± 8.2	4.3 ± 3.6	8.6 ± 7.0

(NA): Not Available.

**Table 5:** Retrovirus genera and type of species affected.

Classification of retroviruses		
Genus	Species	Examples
Alpharetrovirus	Birds	Avian leukosis viruses (AVL), Rous sarcoma virus (RSV)
Betaretrovirus	Mice, primates, and sheep	Mouse mammary tumor virus (MMTV), Mason-Pfizer monkey virus (MPMV), and Jaagsiekte sheep retrovirus (JSRV)
Gammaretrovirus	Mice, cats, primates, and birds	Murine leukemia virus (MLV), feline leukemia viruses, gibbon ape leukemia virus, reticuloendotheliosis virus, and xenotropic murine retrovirus (xMRV)
Deltaretrovirus	Cattle and primates	Bovine leukemia virus, human T-lymphotropic virus
Epsilonretrovirus	Fish	Walleye dermal sarcoma virus
Lentivirus	Primates, sheep, cats, and horses	Human immunodeficiency virus (HIV), simian immunodeficiency virus (SIV), Maedi/visna virus, feline immunodeficiency virus (FIV), and equine infectious anemia virus
Spumavirus	Primates, cats, and cattle	Human foamy virus (HFV), simian foamy virus (SFV), feline foamy virus, and bovine foamy virus

**Table 6:** Gene expression analysis of the spleen tissues were used to investigate the centriole-centrosome pathway for infected versus control mice. *tacc3* showed the highest upregulation with 30.13-fold increase, much more than our previous report [4]. *tpx2* and *p53* showed significant downregulation. During this investigation, *aurka* did not show significant upregulation. This is due to the variation in this group of six mice used in this study. However, a trend of upregulation of *aurka* is evident in 3 mice in column 2.

Gene expression						
Mouse ID	*tacc3*	*aurka*	*ndel1 *	*tpx2 *	*p53 *	*rhamm *
Control 1	(NA)	(NA)	1.18	(NA)	(NA)	(NA)
Control 2	(NA)	(NA)	0.17	(NA)	0.45	0.56
Control 3	2.32	0.8	0.42	0.91	0.66	0.75
Control 4	0.37	0.96	2.78	1.85	1.95	1.53
Control 5	0.85	0.96	0.57	1.19	0.93	1.17
Control 6	0.64	1.52	0.09	0.17	(NA)	(NA)
Control 7	0.82	0.76	1.81	0.88	(NA)	(NA)
SEM	**0.34**	**0.14**	**0.37**	**0.27**	**0.33**	**0.22**
Normalized avg. fold Exp.	**1.00**	**1.00**	**1.00**	**1.00**	**1.00**	**1.00**

Lymphoma 1	11.25	1.25	0.14	0.15	0.26	1.63
Lymphoma 2	(NA)	0.44	0.12	0.2	0.13	0.67
Lymphoma 3	(NA)	0.4	0.25	0.11	0.15	1.63
Lymphoma 4	53.53	2.41	0.2	0.15	0.71	0.88
Lymphoma 5	53.16	1.87	0.24	0.15	0.6	0.9
Lymphoma 6	2.57	(NA)	0.09	0.15	0.07	0.85
Avg Fold Exp.	**30.13**	**1.27**	**0.17**	**0.15**	**0.32**	**1.09**
SEM	**13.52**	**0.39**	**0.03**	**0.01**	**0.11**	**0.17**
*P* ≤ 0.05	**0.04**	**0.53**	**0.07**	**0.01**	**0.05**	**0.75**

(NA): data not available.
